# Urine Gram Stain in the Early Detection of an Enterovesical Fistula

**DOI:** 10.7759/cureus.88038

**Published:** 2025-07-15

**Authors:** Moe Kuroda, Kaku Kuroda, Keiichiro Kita

**Affiliations:** 1 Family Medicine/General Internal Medicine, Toyama University Hospital, Toyama, JPN; 2 Family Medicine/Geriatrics/General Internal Medicine, Toyama University Hospital, Toyama, JPN; 3 General Internal Medicine, Toyama University Hospital, Toyama, JPN

**Keywords:** cecum cancer, entero-vesical fistula, family medicine/general practice, gram stain, point-of-care testing

## Abstract

Enterovesical fistula (EVF) is a rare condition that can be difficult to diagnose due to its nonspecific symptoms, such as recurrent urinary tract infections (UTIs), pneumaturia, and fecaluria. We report a case of EVF in which early diagnosis was facilitated by clinically integrated interpretation of urine Gram stain findings in the context of the patient’s overall presentation. An 89-year-old woman with a history of rheumatoid arthritis and long-term corticosteroid use presented with fever and right knee pain, initially suspected to be a rheumatoid flare. However, synovial fluid analysis was negative for infection or crystals, prompting further evaluation. Urinalysis revealed bacteriuria and leukocyturia, and urine Gram stain demonstrated a polymicrobial pattern with numerous polymorphonuclear leukocytes. Given the patient’s clinical status and lack of common risk factors for polymicrobial colonization, the findings raised suspicion for fecaluria and a possible EVF. A contrast-enhanced computed tomography (CT) scan subsequently confirmed an EVF due to mucinous adenocarcinoma of the cecum invading the bladder. The patient underwent successful ileocecal resection and bladder fistula closure. Although not routinely used to diagnose EVF, urine Gram stain can provide early diagnostic clues when interpreted in real time alongside clinical findings. When polymicrobial patterns are observed in urine, especially in patients with recurrent or atypical UTIs, they may offer a critical diagnostic clue to EVF. In this case, the integration of polymicrobial patterns with bedside assessment led to early suspicion and imaging. The case also highlights how serious infections in older patients may present with atypical or misleading symptoms, emphasizing the need for diagnostic vigilance. This case highlights the potential utility of urine Gram stains as a rapid, cost-effective tool for the early detection of EVF. Clinicians should consider EVF in older adult patients with atypical UTIs, and interpret polymicrobial Gram stain findings in the clinical context to guide timely diagnosis and appropriate management.

## Introduction

Enterovesical fistula (EVF) is an abnormal communication between the bowel and the urinary bladder, most commonly resulting from diverticular disease, colorectal or pelvic malignancy, or inflammatory bowel disease [1,2]. It occurs more frequently in males than in females, with reported male-to-female ratios ranging from 2:1 to 3:1 [3-5]. This disparity is thought to reflect the protective role of the uterus, which anatomically separates the bladder from the adjacent bowel in females, thereby reducing the risk of fistula formation [3]. Diagnosing EVF can be challenging due to a lack of guidelines for diagnosis and its often nonspecific symptoms, such as pneumaturia, fecaluria, and recurrent urinary tract infections (UTIs) [2,6,7]. Although imaging tests, including computed tomography (CT) and magnetic resonance imaging (MRI), are currently considered helpful diagnostic tools [6,8], early clinical suspicion remains key. Early and accurate diagnosis of EVF is crucial for timely intervention and improved patient outcomes, as it enables targeted preoperative care, reducing surgical complexity, and preventing avoidable complications [2,9,10]. In this report, we present a rare case in which a urine Gram stain provided an early diagnostic clue for EVF, emphasizing the value of simple, cost-effective tests in guiding further evaluation.

## Case presentation

An 89-year-old woman with a history of rheumatoid arthritis (RA) presented to the emergency department due to fever and knee pain. She had been diagnosed with RA a year earlier and has received a daily dose of 2 mg prednisone. Vital signs were unremarkable except for a body temperature of 38.1℃. On physical examination, the right knee exhibited tenderness, redness, swelling, and warmth. Abdominal examination yielded benign results. Synovial fluid analysis of the right knee revealed no monosodium urate or calcium pyrophosphate dihydrate crystals. The Gram stain of the synovial fluid showed no evidence of bacteria. Although the knee pain was initially thought to be secondary to an RA flare, the negative synovial fluid analysis prompted further evaluation to identify other potential sources of fever. 

**Figure 1:**
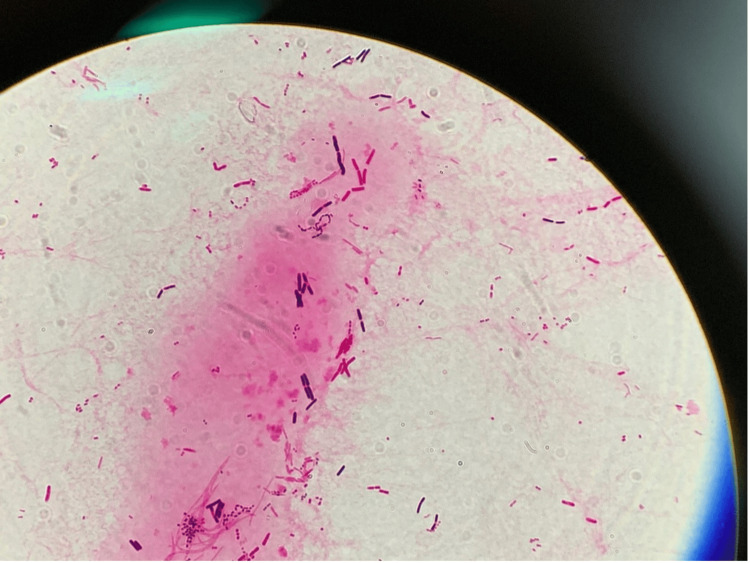
Urine Gram Stain The urine Gram stain revealed a large number of various bacteria with a diverse pattern, covering the entire microscopic field.

**Figure 2:**
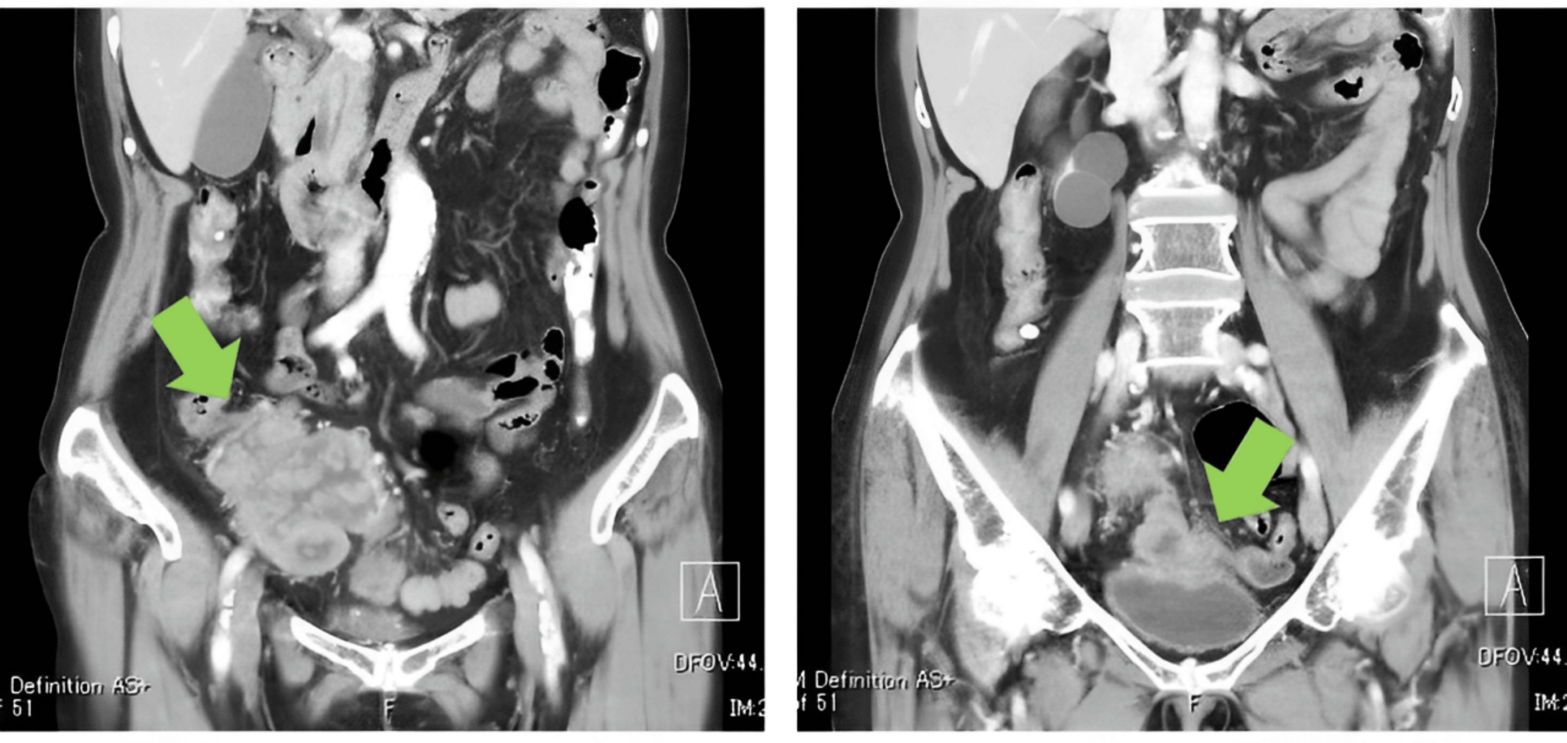
A contrast-enhanced CT scan A contrast-enhanced CT scan demonstrated a fistulous connection between the bladder and the cecum, indicative of an enterovesical fistula (EVF) caused by a malignant tumor (green arrow).

A chest X-ray and urinalysis were performed. The chest X-ray revealed no acute findings, while the urinalysis report by the laboratory noted bacteriuria and leukocyturia with a polymicrobial pattern. Because the patient was not at high risk for polymicrobial urinary colonization - such as in bedridden individuals or those with indwelling catheter use - the clinical team directly reviewed the Gram stain findings. It demonstrated a mixture of gram-negative and gram-positive rods, suggestive of fecaluria (Figure 1). Based on these Gram stain findings, an EVF was suspected. A contrast-enhanced CT scan of the chest, abdomen, and pelvis confirmed the diagnosis, revealing an EVF caused by a malignant tumor (Figure 2A, 2B). The patient was referred to the surgery team and underwent an ileocecal resection along with a simple closure of the bladder fistula. Pathology revealed mucinous adenocarcinoma of the cecum with bladder infiltration of the tumor, and fistula formation.

## Discussion

Diagnosing EVF can be challenging and often takes months after the initial symptoms appear [11]. Asymptomatic bacteriuria is common in older adult women, and not all patients require treatment [12]. However, polymicrobial patterns on microscopic examination, especially those suggesting fecaluria, can serve as a crucial indicator for earlier diagnosis of EVF [7]. 

In our case, the identification of a polymicrobial pattern with polymorphonuclear leukocytes on a urine Gram stain, interpreted in real time by the clinical team in the context of the patient’s symptoms, medical history, and unexplained fever, raised strong suspicion for fecaluria and led to the early diagnosis of EVF. While urine Gram staining is not routinely used for EVF detection, this case illustrates its potential diagnostic value when interpreted promptly and thoughtfully. Polymicrobial findings, especially those involving a mixture of gram-negative and gram-positive rods, should raise suspicion of enteric contamination. 

Several studies from Japan have reported that point-of-care Gram staining by physicians can provide immediate insights and facilitate rapid clinical decision-making [13-15]. In this case, such integration enabled early recognition of fecaluria and a timely CT scan, which confirmed an EVF caused by invasive cecal cancer. This integration is essential not only for quickly identifying unexpected diagnostic clues but also for avoiding inappropriate empirical antibiotic use and promoting more targeted antimicrobial therapy[13-15]. Regardless of who performs the Gram stain, clinicians should consider enteric contamination when polymicrobial urine patterns are observed or reported. When such findings occur-especially in patients with recurrent UTIs or atypical systemic symptoms-further imaging should be pursued to evaluate for enterovesical communication.

Additionally, patient age and comorbidities can influence symptom presentation and delay diagnosis. Older adults, particularly those with chronic conditions such as RA or those receiving long-term corticosteroids, may present with vague or atypical symptoms even when serious infections are present [16,17]. In this case, the patient’s initial complaint of joint pain and fever could have been misattributed to an RA flare. However, Gram stain findings suggestive of fecaluria pointed to a UTI and ultimately led to the diagnosis of an EVF. This case underscores the need to include UTI in the differential diagnosis of older adult women and to interpret Gram stain findings with care.

## Conclusions

Urine Gram stain, when carefully interpreted in the clinical context, can offer early and valuable clues in the diagnosis of EVF. This case highlights the importance of integrating laboratory findings with clinical judgment in real time. Clinicians should maintain awareness of the significance of polymicrobial urine patterns and their diagnostic value in detecting undiagnosed EVF. Additionally, clinicians should recognize that older adults with comorbidities may present with atypical symptoms of UTIs, emphasizing the importance of a broad differential and thoughtful interpretation of Gram stain findings.

## References

[1] Daniels IR, Bekdash B, Scott HJ, Marks CG, Donaldson DR (2002). Diagnostic lessons learnt from a series of enterovesical fistulae. Colorectal Dis.

[2] Kavanagh D, Neary P, Dodd JD, Sheahan KM, O'Donoghue D, Hyland JM (2005). Diagnosis and treatment of enterovesical fistulae. Colorectal Dis.

[3] Widia F, Firman M, Irdam GA, Syaiful RA (2022). A six years' experience with 41 cases of enterovesical fistula in a tertiary national hospital in Indonesia: a retrospective study. Ann Med Surg (Lond).

[4] Granieri S, Sessa F, Bonomi A (2021). Indications and outcomes of enterovesical and colovesical fistulas: systematic review of the literature and meta-analysis of prevalence. BMC Surg.

[5] Misiak M, Dworak M, Wyszomirska M, Kurt M, Walędziak M, Różańska-Walędziak A (2023). Gynecological fistulae-has anything changed in the diagnosis and treatment over the last decade? A narrative literature review. Medicina (Kaunas).

[6] Xu R, Vaughan A, Fagan M, Schumacher DP, Wekullo V, Gehrke B (2023). Colovesical fistula in men with chronic urinary tract infection: a diagnostic challenge. Cleve Clin J Med.

[7] Raymond PL, Gibler WB (1989). Detection of colovesical fistula in the emergency department: report of a case. Am J Emerg Med.

[8] Golabek T, Szymanska A, Szopinski T, Bukowczan J, Furmanek M, Powroznik J, Chlosta P (2013). Enterovesical fistulae: aetiology, imaging, and management. Gastroenterol Res Pract.

[9] Larsen A, Bjerklund Johansen TE, Solheim BM, Urnes T (1996). Diagnosis and treatment of enterovesical fistula. Eur Urol.

[10] Karkhanis P, Patel A, Galaal K (2012). Urinary tract fistulas in radical surgery for cervical cancer: the importance of early diagnosis. Eur J Surg Oncol.

[11] Scozzari G, Arezzo A, Morino M (2010). Enterovesical fistulas: diagnosis and management. Tech Coloproctol.

[12] Nicolle LE, Gupta K, Bradley SF (2019). Clinical practice guideline for the management of asymptomatic bacteriuria: 2019 update by the Infectious Diseases Society of America. Clin Infect Dis.

[13] Yoshimura J, Ogura H, Oda J (2023). Can Gram staining be a guiding tool for optimizing initial antimicrobial agents in bacterial infections?. Acute Med Surg.

[14] Yodoshi T, Matsushima M, Taniguchi T, Kinjo Kinjo (2019). Utility of point-of-care Gram stain by physicians for urinary tract infection in children ≤36 months.. Medicine (Baltimore).

[15] Taniguchi T, Tsuha S, Shiiki S, Narita M (2020). Point-of-care cerebrospinal fluid Gram stain for the management of acute meningitis in adults: a retrospective observational study.. Ann Clin Microbiol Antimicrob.

[16] George MD, Baker JF, Winthrop K (2020). Risk for serious infection with low-dose glucocorticoids in patients with rheumatoid arthritis: a cohort study. Ann Intern Med.

[17] Listing J, Gerhold K, Zink A (2013). The risk of infections associated with rheumatoid arthritis, with its comorbidity and treatment. Rheumatology (Oxford).

